# Incidence, survival, and associated factors estimation in osteosarcoma patients with lung metastasis: a single-center experience of 11 years in Tianjin, China

**DOI:** 10.1186/s12885-023-11024-9

**Published:** 2023-06-05

**Authors:** Chao Zhang, Haixiao Wu, Guijun Xu, Yao Xu, Wenjuan Ma, Zhijun Li, Jin Zhang

**Affiliations:** 1 Department of Bone and Soft Tissue Tumors, Key Laboratory of Cancer Prevention and Therapy, Tianjin Medical University Cancer Institute and Hospital, National Clinical Research Center for Cancer, Tianjin’s Clinical Research Center for Cancer, Tianjin, 300000 China; 2 Department of Orthopedics, Tianjin Hospital, Tianjin, 300211 China; 3 Department of Breast Imaging, Key Laboratory of Cancer Prevention and Therapy, Tianjin Medical University Cancer Institute and Hospital, National Clinical Research Center for CancerTianjin’s Clinical Research Center for Cancer, Tianjin, 300000 China; 4 Department of Radiology, Key Laboratory of Cancer Prevention and Therapy, Tianjin Medical University Cancer Institute and Hospital, National Clinical Research Center for Cancer, Tianjin’s Clinical Research Center for Cancer, Tianjin, 300000 China

**Keywords:** Osteosarcoma, Survival, Pulmonary metastasis

## Abstract

**Background:**

Osteosarcoma is the most common primary malignant bone tumor. The current study was conducted to describe the general condition of patients with primary osteosarcoma in a single cancer center in Tianjin, China and to investigate the associated factors in osteosarcoma patients with lung metastasis.

**Methods:**

From February 2009 to October 2020, patients from Tianjin Medical University Cancer Institute and Hospital, China were retrospectively analyzed. The Kaplan–Meier method was used to evaluate the overall survival of osteosarcoma patients. The Cox proportional hazard regression analysis was performed to analyze the prognostic factors of all osteosarcoma patients and those patients with lung metastasis, respectively. Furthermore, risk factors for developing lung metastasis were identified in synchronous lung metastasis (SLM) and metachronous lung metastasis (MLM) patients.

**Results:**

A total of 203 patients were involved and 150 patients were successfully followed up for survival status. The 5-year survival rate of osteosarcoma was 70.0% and the survival months for patients with SLM and MLM were 33.3 ± 12.6 and 45.8 ± 7.4 months, respectively. The presence of lung metastasis was one of the independent prognostic factors for prognosis of osteosarcoma. In patients with lung metastasis, twenty-one (10.3%) showed lung metastasis at the diagnosis of osteosarcoma and 67 (33%) were diagnosed with lung metastases during the later course. T3 stage (OR = 11.415, 95%CI 1.362–95.677, *P* = *0.025*) and bone metastasis (OR = 6.437, 95%CI 1.69–24.51, *P* = *0.006*) were risk factors of SLM occurrence. Bone metastasis (OR = 1.842, 95%CI 1.053–3.224, *P* = *0.032*), good necrosis (≥ 90%, OR = 0.032, 95%CI 0.050–0.412, *P* < *0.001*), elevated Ki-67 (OR = 2.958, 95%CI 1.098–7.969, *P* = *0.032*) and elevated LDH (OR = 1.791, 95%CI 1.020–3.146, *P* = *0.043*) were proved to be independent risk factors for developing MLM.

**Conclusion:**

The overall survival, prognostic factors and risk factors for lung metastasis in this single center provided insight about osteosarcoma management.

**Supplementary Information:**

The online version contains supplementary material available at 10.1186/s12885-023-11024-9.

## Background

Osteosarcoma is the most common primary malignant bone tumor in young adult, the prevalence was reported to be 8–11 per million people per year [1]. Since the comprehensive treatment strategy by chemotherapy and surgery, the 5-year overall survival (OS) rate has been significantly improved [2, 3].

Distant metastasis, especially the lung metastasis, has been a serious issue in osteosarcoma management. To facilitate related studies and improve outcomes of osteosarcoma, four clinical oncology groups in European and American collaborated to construct the EURAMOS (European and American Osteosarcoma Studies) group [4]. Through international, collaborative randomized, controlled trials (RCTs), their first study (EURAMOS-1) recruited a total of 2260 patients with resectable high-grade osteosarcoma across 17 countries from 2005 to 2011 [4]. After a median of 54 months follow-up, the 5-year event-free survival (EFS) and overall survival rates were reported to be 59.0% and 71.0%, respectively [5]. Specifically, patients with lung metastasis had a 2.34-fold higher risk of death when compared with those without lung metastasis [5]. In fact, several studies have reported the negative effect of lung metastasis on survival in osteosarcoma [6, 7]. The 5-year overall survival was just 20–30% in patients with lung metastasis [2]. A previous study, based on 1,408 patients with osteosarcoma in Surveillance, Epidemiology and End Results (SEER) database, reported a total of 238 patients (16.9%) with lung metastases at diagnosis [8]. Similarly, the latest study showed that around 14% osteosarcoma patients were with lung metastasis at diagnosis and the indeterminate nodules in lung can turn into the metastatic disease at a median time of 5.3 months [9]. Lung metastasis has become the focus in osteosarcoma in recent years.

Under the consideration of poor prognosis in lung metastatic patients, computerized tomography (CT) of the chest was recommended as the routine examination for patients with osteosarcoma, especially for those with suspicious lung lesions [9, 10]. However, the differential diagnosis of benign and malignant in both the nodules (<5mm) and indeterminate nodules has been treated as the challenging issue among bone oncology surgeons [9, 11, 12]. In the osteosarcoma patients with high risk of metastasis, PET-CT was recommended for its high sensitivity (90%). Thus, risk evaluation on metastasis in osteosarcoma at diagnosis and during later course is important. The previous study reported that the large tumor size was associated with the higher odds of lung metastasis occurrence. Patients with tumor size larger than 371cm^3^ showed a probability of 69% to suffer lung metastasis, compared with 34% in those with the smaller tumor size [13]. Axial location, tumor size >10 cm, higher N stage and bone metastasis presence were reported to be significant risk factors of lung metastasis in osteosarcoma [8]. These findings were valuable to identify the high-risk patients.

After widely literatures reviewing, most studies on osteosarcoma were performed based on Caucasian population. In China, with the development of Chinese Society of Clinical Oncology, the standardized treatment has been widely introduced and performed. As the first established department of bone and soft tissue sarcoma in China, we have the advantage to treat large population of osteosarcoma with the standardized chemotherapy and surgery by the same multidisciplinary team. Thus, we summarized our experience in the past ten years. Based on the single-center data, the survival and prognostic factors of patients with osteosarcoma were investigated. The incidences of both synchronous and metachronous lung metastasis were evaluated and the risk factors of lung metastasis were explored.

## Methods

### Patient selection

This retrospective study was approved by the institutional research ethics committee of Tianjin Medical University Cancer Institute and Hospital (NO. bc2021011). Based on medical records from February 2009 to October 2020, patients with historically diagnosed osteosarcoma were selected and followed by phone/clinic until December 2020. The inclusion criteria were listed as following: (a) historically diagnosed as primary osteosarcoma; (b) complete basic information; (c) clear evidence of lung metastasis. Patients were excluded if the survival status or lung metastasis was not available. Patients who cannot be followed were also excluded.

### Variables used in current study

Variables were involved as following: age (≤ 18 years, 19–40 years, ≥ 41 years), gender (male and female), tumor site (upper limb, lower limb, spine/pelvis), surgery (no, salvage, amputation and unknown), necrosis (Huvos I-II < 90%, Huvos III-IV ≥ 90%), alkaline phosphatase (ALP) level (normal, elevated in one time than the upper limitation, elevated more than two times), lactic dehydrogenase (LDH) level (normal and elevated), bone metastasis (no, yes) and lung metastasis (no, synchronous, metachronous). The T stage and N stage in the present study were defined according to TNM Staging System for Bone in American Joint Committee on Cancer (AJCC), which was listed in supplementary Table 1. Lung metastasis was diagnosed by pathological examination or chest CT according to the standard described by Tsoi KM [9]. For patients who received biopsy, the diagnosis was determined based on pathologic findings. Moreover, the increase of lung nodule’s size more than 25% or the appearance of new nodule during follow-up chest CT were diagnosed with lung metastasis. Synchronous lung metastasis (SLM) was defined as lung metastasis diagnosed at the initial osteosarcoma diagnosis while metachronous lung metastasis (MLM) was defined as the occurrence of lung metastasis in patients’ later course.

### Treatment

As for high grade localized osteosarcoma, neoadjuvant chemotherapy combined with surgery and adjuvant chemotherapy were performed according to NCCN guidelines. The standard first-line neoadjuvant chemotherapy in our department is cisplatin, doxorubicin, and high-dose methotrexate (MAP) regimen. For patients with good histologic response to neoadjuvant chemotherapy, wide excision and limb salvage were performed, followed by another four cycles of the same chemotherapy regimen after surgery. For patients with recurrent or refractory disease, the combination of etoposide and ifosfamide (IE) was used. Besides, the IE regimen was performed for patients who received MAP regimen previously.

During 11 years, to reconstruct the large bony defect, several kinds of surgeries were performed, including joint preserving surgery, tumor-devitalized autograft, and 3D printing implant. The prosthetic replacement was commonly performed on patients with osteosarcoma in upper and lower limbs. The tumor-devitalized autograft was performed with frozen autograft technique on the cases with unsatisfactory margin. The 3D printing implant was performed to preserve the joint function in children.

### Statistics

The quantitative data were described as mean ± standard deviation (SD) and categorical data were presented as the number and the percentage (N, %). Pearson chi-square test was used to evaluate the difference between categorical variables. The overall survival was defined as the time from the diagnosis of osteosarcoma to all causes of death, which was analyzed using the Kaplan–Meier method. The survival difference between groups was tested by the Log-rank test and prognostic factors of osteosarcoma were identified by the Cox proportional hazard regression analysis.

For patients with lung metastasis, the time period from the diagnosis of lung metastasis to all causes of death was recorded and related prognostic factors were explored using the Cox proportional hazard regression analysis. Further analyses were conducted to explore the risk factors for developing lung metastasis in different pattern of lung metastasis (SLM and MLM). Initially, patients with MLM were deemed as no lung metastasis at the diagnosis of osteosarcoma and the Logistic regression analysis was used to identify the risk factors for developing SLM. When exploring the risk factors of MLM, patients with SLM were excluded from the analysis and the Cox proportional hazard regression analysis was performed.

Two-sided *P* < 0.05 was considered as statistically significant. Variables with *P*-value < 0.05 in the univariate regression analysis were further analyzed using a multivariate regression analysis. All statistical analyses were performed using SPSS 22.0 (IBM Corporation, NY, USA).

## Results

### Characteristics and survival outcome of patients with osteosarcoma

Patients with osteosarcoma were reviewed and followed by phone/clinic with the follow-up time ranged from 2 to 144 months. Eventually, a total of 203 patients were identified with the clear status of lung metastasis and the demographic and clinical characteristics were described in Table 1. The average age at the diagnosis of osteosarcoma was 22.8 ± 14.2 (5–77) years. The historical types of osteosarcoma tumor were described as following: osteosarcoma NOS (*N* = 88), conventional osteosarcoma (*N* = 89), telangiectatic osteosarcoma (*N* = 7), small cell osteosarcoma (*N* = 3), low-grade central osteosarcoma (*N* = 4), parosteal osteosarcoma (*N* = 8) and periosteal osteosarcoma (*N* = 4). Since patients with low-grade tumors (low-grade central osteosarcoma, parosteal osteosarcoma and periosteal osteosarcoma) do not undergo chemotherapy routinely, they were excluded from chemotherapy variable in Table 1. A total of eighty-eight patients (43.3%) were diagnosed with lung metastasis, among which twenty-one patients (10.3%) were diagnosed with SLM and sixty-seven patients (33.0%) were diagnosed with MLM.

**Table 1:** Demographic and clinical characteristics of the included patients

Variables	No Lung Mets	Synchronous Lung Mets	Metachronous Lung Mets	χ2	*P-value*
Age (year)					
≤ 18	57 (49.6%)	11 (52.4%)	39 (58.2%)	1.367	*0.850*
19–40	43 (37.4%)	7 (33.3%)	21 (31.3%)		
≥ 41	15 (13.0%)	3 (14.3%)	7 (10.4%)		
Gender					
Male	64 (55.7%)	13 (61.9%)	44 (65.7%)	1.817	*0.403*
Female	51 (44.3%)	8 (38.1%)	23 (34.3%)		
Tumor site					
Upper limb	12 (10.4%)	4 (19.0%)	9 (13.4%)	4.218	*0.377*
Lower limb	100 (87.0%)	15 (71.4%)	54 (80.4%)		
Spine-pelvis	3 (2.6%)	2 (9.5%)	4 (6.0%)		
Stage T					
T1	43 (37.4%)	7 (33.3%)	23 (36.0%)	7.873	*0.248*
T2	68 (59.1%)	12 (57.1%)	42 (60.1%)		
T3	1 (0.9%)	2 (9.5%)	1 (2.0%)		
Unknown	3 (2.6%)	0 (0%)	1 (2.0%)		
Stage N					
N0	81 (70.4%)	14 (66.7%)	44 (65.7%)	8.405	*0.078*
N1	0 (0%)	0 (0%)	4 (6.0%)		
Unknown	34 (29.6%)	7 (33.3%)	19 (28.4%)		
Bone Mets					
No	100 (87.0%)	13 (61.9%)	46 (68.7%)	12.071	*0.002*
Yes	15 (13.0%)	8 (38.1%)	21 (31.3%)		
Surgery					
No	7 (6.1%)	10 (47.6%)	3 (4.5%)	47.557	< *0.001*
Salvage	64 (55.7%)	5 (23.8%)	31 (46.3%)		
Amputation	31 (27.0%)	4 (19.0%)	31 (46.3%)		
Unknown	13 (11.3%)	2 (9.5%)	2 (3.0%)		
Chemotherapy					
No	0 (0%)	3 (15.0%)	0 (0%)	31.355	< *0.001*
Yes	87 (86.1%)	16 (80.0%)	64 (97.0%)		
Unknown	14 (13.9%)	1 (5.0%)	2 (3.0%)		
Necrosis					
< 90%	26 (22.6%)	5 (23.8%)	34 (50.7%)	26.393	< *0.001*
≥ 90%	27 (23.5%)	0 (0%)	4 (6.0%)		
Unknown	62 (53.9%)	16 (76.2%)	29 (43.3%)		
Ki-67					
< 50%	35 (30.4%)	3 (14.3%)	8 (11.9%)	9.617	*0.047*
≥ 50%	12 (10.4%)	2 (9.5%)	10 (14.9%)		
Unknown	68 (59.1%)	16 (76.2%)	49 (73.1%)		
LDH					
Normal	89 (77.4%)	13 (61.9%)	41 (61.2%)	6.155	*0.046*
Elevated	26 (22.6%)	8 (38.1%)	26 (38.8%)		
ALP					
Normal	44 (38.3%)	8 (38.1%)	19 (28.4%)	6.172	*0.187*
Within one time	40 (34.8%)	6 (28.6%)	18 (26.9%)		
More than two times	31 (27.0%)	7 (33.3%)	30 (44.8%)		

*Abbreviations*: *Mets* Metastases, *ALP* Alkaline phosphatase, *LDH* Lactate dehydrogenase

**Fig. 1:**
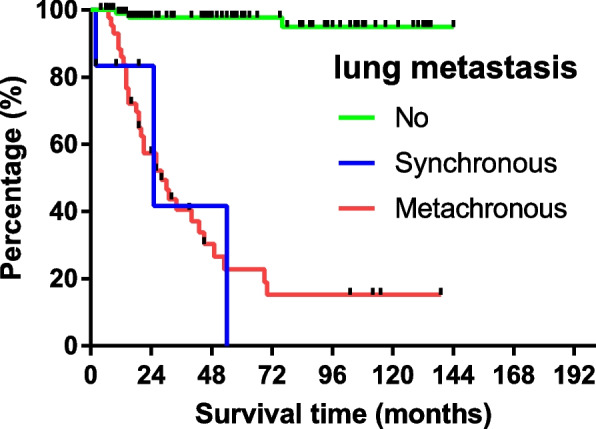
Survival curves for patients with or without lung metastasis

A total of 150 patients were in active follow-up and theoverall survival (OS) of all patients was 104.7 ± 5.4 [95%CI Confidence interval (CI) 94.1–115.3] months. The 1-, 3-, 5-year survival rate was 94.4%, 77.3% and 70.0%, respectively. For patients without lung metastasis, the average OS was up to 139.2 ± 2.8 (95%CI 133.7–144.6) months. The survival was worse in patients with lung metastasis: 33.3 ± 12.6 (95%CI 8.6–57.9) months for SLM patients and 45.8 ± 7.4 (95%CI 31.3–60.3) months for MLM patients, respectively. The survival curve of different lung metastasis pattern was illustrated in Fig. 1. More survival outcome of patients within different variables and the statistical results were shown in Table 2.

**Table 2:** Overall survival of osteosarcoma patients in different variables

Variables	3-year OSR	5-year OSR	AOS (95%CI) months	χ2	*P-value*
Age (year)					
≤ 18	72.9%	59.6%	91.9 ± 7.6 (77.0–106.8)	1.989	*0.370*
19–40	83.3%	80.6%	113.1 ± 7.7 (98.0–128.1)		
≥ 41	73.6%	NA	100.4 ± 12.9 (75.2–125.6)		
Gender					
Male	68.8%	64.3%	96.7 ± 7.6 (81.8–111.6)	2.633	*0.105*
Female	87.6%	76.9%	108.1 ± 6.8 (94.8–121.5)		
Tumor site					
Upper limb	66.7%	NA	85.7 ± 13.0 (60.4–111.1)	6.522	*0.038*
Lower limb	80.0%	71.8%	106.9 ± 5.7 (95.7–118.1)		
Spine-pelvis	NA	NA	26.4 ± 5.5 (15.7–37.2)		
Stage T					
T1	84.0%	69.4%	98.2 ± 8.3 (82.0–114.5)	1.683	*0.641*
T2	73.5%	NA	98.3 ± 6.4 (85.8–110.9)		
T3	NA	NA	16.0 ± 3.3 (9.6–22.4)		
Unknown	NA	NA	110.5 ± 29.0 (53.6–167.4)		
Stage N					
N0	78.5%	69.5%	103.4 ± 6.1 (91.5–115.3)	12.595	*0.002*
N1	0	NA	19.8 ± 6.9 (6.2–33.3)		
Unknown	79.8%	75.8%	106.7 ± 10.4 (86.4–127.0)		
Bone Mets					
No	89.7%	79.9%	116.0 ± 5.5 (105.3–126.7)	12.890	< *0.001*
Yes	68.3%	49.1%	68.8 ± 10.0 (49.2–88.3)		
Lung Mets					
No	NA	NA	139.2 ± 2.8 (133.7–144.6)	89.029	< *0.001*
SLM	41.7%	0	33.3 ± 12.6 (8.6–57.9)		
MLM	40.5%	22.8%	45.8 ± 7.4 (31.3–60.3)		
Surgery					
No	NA	NA	57.4 ± 16.6 (24.9–89.9)	9.748	*0.021*
Salvage	75.6%	69.6%	101.6 ± 6.7 (88.4–114.7)		
Amputation	80.7%	70.2%	101.2 ± 9.1 (83.5–119.0)		
Unknown	NA	NA	78.3 ± 5.5 (67.6–89.0)		
Chemotherapy					
No	NA	NA	NA	1.692	*0.429*
Yes	75.7%	68.0%	NA		
Unknown	NA	NA	NA		
Necrosis					
< 90%	69.3%	51.5%	80.0 ± 8.3 (63.6–96.4)	10.297	*0.006*
≥ 90%	NA	NA	130.0 ± 4.9 (120.5–139.6)		
Unknown	76.2%	72.6%	108.7 ± 7.6 (93.8–123.7)		
Ki-67					
< 50%	77.0%	NA	92.5 ± 9.7 (73.5–111.5)	2.399	*0.301*
≥ 50%	NA	NA	98.8 ± 9.7 (79.8–117.8)		
Unknown	74.5%	NA	99.9 ± 6.4 (87.3–112.6)		
LDH					
Normal	79.7%	70.1%	106.1 ± 6.1 (94.2–118.1)	0.554	*0.456*
Elevated	70.2%	NA	97.8 ± 10.9 (76.5–119.1)		
ALP					
Normal	84.9%	78.7%	113.8 ± 7.9 (98.3–129.3)	8.150	*0.017*
Within one time	80.9%	NA	112.7 ± 8.3 (96.5–129.0)		
More than two times	64.0%	51.8%	75.0 ± 9.1 (57.1–92.8)		

Abbreviations: *OSR* Overall survival rate, *AOS* Average overall survival, *Mets* Metastases, *SLM* Synchronous lung metastasis, *MLM* Metachronous lung metastasis, *ALP* Alkaline phosphatase, *LDH* Lactate dehydrogenase, *NA* Not available

### The prognostic factors of osteosarcoma patients with lung metastasis

The prognostic factors of all patients with active follow-up were identified and illustrated in the Table 3. In multivariate Cox regression analysis, the presence of bone metastasis (HR = 2.447, 95%CI 1.036–5.780, *P* = *0.041*), the presence of lung metastasis (HR = 55.817, 95%CI 12.83–242.837, *P* < *0.001*), salvage (HR = 0.262, 95%CI 0.077–0.887, *P* = *0.031*) and amputation (HR = 0.096, 95%CI 0.024–0.376, *P* = *0.001*) were four independent prognostic factors.

**Table 3:** Identification of the prognostic factors in patients with osteosarcoma (*N* = 150)

Variables	Univariate		Multivariate	
	**HR (95% CI)**	***P value***	**HR (95% CI)**	***P value***
Age (year)				
≤ 18	1.00 (Reference)			
19–40	0.603 (0.295–1.233)	*0.166*		
≥ 41	0.769 (0.264–2.244)	*0.631*		
Gender				
Male	1.00 (Reference)			
Female	0.569 (0.285–1.136)	*0.110*		
Tumor site				
Upper limb	1.00 (Reference)			
Lower limb	0.828 (0.292–2.354)	*0.724*		
Spine-pelvis	3.550 (0.778–16.192)	*0.102*		
Stage T				
T1	1.00 (Reference)			
T2	1.078 (0.543–2.141)	*0.829*		
T3	3.480 (0.443–27.355)	*0.236*		
Unknown	0.793 (0.103–6.089)	*0.824*		
Stage N				
N0	1.00 (Reference)		1.00 (Reference)	
N1	6.630 (1.962–22.401)	*0.002*	2.675 (0.732–9.772)	*0.137*
Unknown	1.041 (0.484–2.240)	*0.918*	2.587 (0.978–6.846)	*0.055*
Bone Mets				
No	1.00 (Reference)		1.00 (Reference)	
Yes	3.094 (1.613–5.934)	*0.001*	2.447 (1.036–5.780)	*0.041*
Lung Mets				
No	1.00 (Reference)		1.00 (Reference)	
Yes	37.249 (11.330–122.462)	< *0.001*	55.817 (12.83–242.837)	< *0.001*
Surgery				
No	1.00 (Reference)		1.00 (Reference)	
Salvage	0.244 (0.081–0.728)	*0.011*	0.262 (0.077–0.887)	*0.031*
Amputation	0.283 (0.092–0.870)	*0.028*	0.096 (0.024–0.376)	*0.001*
Unknown	0.082 (0.009–0.740)	*0.026*	0.076 (0.007–0.793)	*0.031*
Chemotherapy				
No	1.00 (Reference)			
Yes	NA	*NA*		
Unknown	NA	*NA*		
Necrosis				
< 90%	1.00 (Reference)		1.00 (Reference)	
≥ 90%	0.085 (0.011–0.633)	*0.016*	1.273 (0.124–13.096)	*0.839*
Unknown	0.568 (0.294–1.097)	*0.092*	1.003 (0.463–2.176)	*0.993*
Ki-67				
< 50%	1.00 (Reference)			
≥ 50%	1.494 (0.334–6.678)	*0.599*		
Unknown	2.187 (0.758–6.308)	*0.148*		
LDH				
Normal	1.00 (Reference)			
Elevated	1.316 (0.636–2.721)	*0.459*		
ALP				
Normal	1.00 (Reference)		1.00 (Reference)	
Within one time	1.662 (0.820–3.369)	*0.969*	1.123 (0.373–3.377)	*0.837*
More than two times	2.453 (1.154–5.214)	*0.020*	1.38 (0.524–3.63)	*0.514*

*Abbreviations*: *NA* Not available, *HR* Hazard ratio, *CI* Confidence interval, *Mets* Metastases, *ALP* Alkaline phosphatase, *LDH* Lactate dehydrogenase

The aforementioned results in Fig. 1 and Table 2 indicated that patients with various patterns of lung metastasis presented different survival outcome. To further explore the differences between groups and identify prognostic factors in osteosarcoma patients with lung metastasis, patients without lung metastasis at the last follow-up were excluded. At last, a total of fifty patients were included into the present analysis. As shown in supplementary Table 2, no independent prognostic factor was identified in our analysis.

### Risk factors for developing lung metastasis in osteosarcoma

As shown in Table 4, the variables associated with SLM in the univariate Logistic regression included T3 stage [Odds ratio (OR) = 9.429, 95%CI 1.144–77.70, *P* = *0.037*] and the presence of bone metastasis (OR = 5.882, 95%CI 1.56–22.14, *P* = *0.009*). After stratified by multivariate analysis, T3 stage (OR = 11.415, 95%CI 1.362–95.677, *P* = *0.025*) and bone metastasis (OR = 6.437, 95%CI 1.69–24.51, *P* = *0.006*) were proved to be two risk factors of SLM occurrence.

**Table 4:** Identification of the risk factors for development synchronous lung metastasis in patients with osteosarcoma

Variables	Univariate		Multivariate	
	**OR (95% CI)**	***P value***	**OR (95% CI)**	***P value***
Age (year)				
≤ 18	1.00 (Reference)			
19–40	0.955 (0.352–2.592)	*0.927*		
≥ 41	1.190 (0.306–4.628)	*0.802*		
Gender				
Male	1.00 (Reference)			
Female	0.898 (0.355–2.274)	*0.821*		
Tumor site				
Upper limb	1.00 (Reference)			
Lower limb	0.511 (0.155–1.687)	*0.271*		
Spine-pelvis	1.500 (0.224–10.036))	*0.676*		
Stage T				
T1	1.00 (Reference)		1.00 (Reference)	
T2	1.029 (0.386–2.743)	*0.955*	1.021 (0.374–2.79)	*0.968*
T3	9.429 (1.144–77.702)	*0.037*	11.415 (1.362–95.677)	*0.025*
Unknown	NA	*NA*	NA	*NA*
Bone Mets				
No	1.00 (Reference)		1.00 (Reference)	
Yes	5.882(1.563–22.143)	*0.009*	6.437 (1.69–24.513)	*0.006*
LDH				
Normal	1.00 (Reference)			
Elevated	1.538 (0.602–3.929)	*0.368*		
ALP				
Normal	1.00 (Reference)			
Within one time	0.815 (0.267–2.489)	*0.719*		
More than two times	0.904 (0.309–2.644)	*0.853*		

*Abbreviations*: *NA* Not available, *OR* Odds ratio, *CI* Confidence interval, *Mets* Metastases, *ALP* Alkaline phosphatase, *LDH* Lactate dehydrogenase

**Fig. 2:**
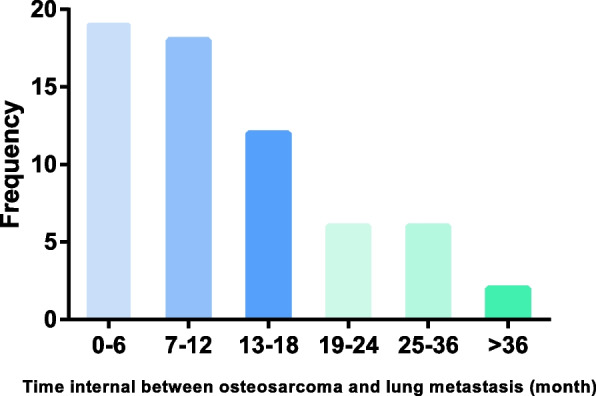
The interval between the diagnosis of osteosarcoma and the diagnosis of lung metastasis in patients with metachronous lung metastasis

For MLM, the information of the interval from osteosarcoma diagnosis to lung metastasis was available in 63 patients. The median internal time was 11 (2–99) months and the distribution of MLM was illustrated in Fig. 2. A total of 37 (58.7%) MLM patients were found in the first year after the diagnosis of osteosarcoma, 18 (28.6%) patients in the second year, and 8 (12.7%) patients in the later time. Chemotherapy was routinely scheduled as previously described for these patients. Noteworthily, metastasectomy of the pulmonary lesion was performed in five patients. The detailed information of the five patients was described in supplementary Table 3.

In univariate Cox regression analysis, the presence of bone metastasis (OR = 1.982, 95%CI 1.164–3.373, *P* = *0.012*), good necrosis (≥ 90%, OR = 0.158, 95%CI 0.056–0.448, *P* = *0.001*), elevated Ki-67 (OR = 3.074, 95%CI 1.183–7.987, *P* = *0.021*), elevated LDH (OR = 2.082, 95%CI 1.249–3.470, *P* = *0.005*) and elevated ALP more than two times (OR = 2.262, 95%CI 1.246–4.106, *P* = *0.007*) were associated with the occurrence of MLM. In multivariate analysis, bone metastasis (OR = 1.842, 95%CI 1.053–3.224, *P* = *0.032*), good necrosis (≥ 90%, OR = 0.032, 95%CI 0.050–0.412, *P* < *0.001*), elevated Ki-67 (OR = 2.958, 95%CI 1.098–7.969, *P* = *0.032*) and elevated LDH (OR = 1.791, 95%CI 1.020–3.146, *P* = *0.043*) were proved to be independent risk factors for developing MLM. Details information of the Cox regression analysis were summarized in Table 5.

**Table 5:** Identification of the risk factors for developing metachronous lung metastasis in osteosarcoma patients using the Cox proportional hazard regression analysis

Variables	Univariate		Multivariate	
	**OR (95% CI)**	***P value***	**OR (95% CI)**	***P value***
Age (year)				
≤ 18	1.00 (Reference)			
19–40	0.758 (0.438–1.312)	*0.323*		
≥ 41	0.717 (0.319–1.612)	*0.421*		
Gender				
Male	1.00 (Reference)			
Female	0.631 (0.373–1.067)	*0.086*		
Tumor site				
Upper limb	1.00 (Reference)			
Lower limb	0.891 (0.405–1.963)	*0.775*		
Spine-pelvis	2.024 (0.589–6.958)	*0.263*		
Stage T				
T1	1.00 (Reference)			
T2	1.096 (0.650–1.849)	*0.731*		
T3	2.377 (0.319–17.718)	*0.398*		
Unknown	0.529 (0.071–3.94)	*0.535*		
Stage N				
N0	1.00 (Reference)			
N1	25.026 (6.879–91.043)	*0*		
Unknown	1.106 (0.629–1.944)	*0.727*		
Bone Mets				
No	1.00 (Reference)		1.00 (Reference)	
Yes	1.982 (1.164–3.373)	*0.012*	1.842 (1.053–3.224)	*0.032*
Surgery				
No	1.00 (Reference)			
Salvage	0.731 (0.174–3.075)	*0.669*		
Amputation	1.341 (0.32–5.609)	*0.688*		
Unknown	0.312 (0.044–2.222)	*0.245*		
Chemotherapy				
No	1.00 (Reference)			
Yes	NA	*NA*		
Unknown	NA	*NA*		
Necrosis				
< 90%	1.00 (Reference)		1.00 (Reference)	
≥ 90%	0.158 (0.056–0.448)	*0.001*	0.143 (0.050–0.412)	< *0.001*
Unknown	0.470 (0.281–0.785)	*0.004*	0.515 (0.293–0.904)	*0.021*
Ki-67				
< 50%	1.00 (Reference)		1.00 (Reference)	
≥ 50%	3.074 (1.183–7.987)	*0.021*	2.958 (1.098–7.969)	*0.032*
Unknown	2.249 (1.058–4.779)	*0.035*	2.371 (1.112–5.056)	*0.025*
LDH				
Normal	1.00 (Reference)		1.00 (Reference)	
Elevated	2.082 (1.249–3.470)	*0.005*	1.791 (1.020–3.146)	*0.043*
ALP				
Normal	1.00 (Reference)		1.00 (Reference)	
Within one time	1.222 (0.629–2.371)	*0.554*	1.256 (0.620–2.544)	*0.527*
More than two times	2.262 (1.246–4.106)	*0.007*	1.317 (0.660–2.626)	*0.434*

*Abbreviations*: *NA* Not available, *OR* Odds ratio, *CI* Confidence interval, *Mets* Metastases, *ALP* Alkaline phosphatase, *LDH* Lactate dehydrogenase

## Discussion

In the current study, we summarized our experience from 203 osteosarcoma patients. Based on the cohort, the 5-year survival rate was 70.0%. Such long-term survival reached a promising level, which was better than that in our previous study based on SEER data from 2010 and 2016 [14].

Based on the Cox regression analysis, the presence of bone metastasis and lung metastasis were associated with worse survival in osteosarcoma and the performance of surgery was associated with better survival outcome. Since 1970s, the introduction of chemotherapy significantly improved the survival of osteosarcoma patients. With the afterwards development of the innovated surgeries, the comprehensive treatment from multidisciplinary team was recommended [10]. In a recent meta-analysis, patients after limb-salvage surgery achieved better five-year survival rate than the patients after amputation with neoadjuvant chemotherapy [15]. Thus, the salvage surgery has become the first choice for eligible patients just did as the current study (salvage 52.2% vs. amputation 32%). Chemotherapy and good tumor necrosis were another important issue in the treatment of osteosarcoma [16]. Chemotherapy response showed significant correlation with the long-term survival in osteosarcoma [17, 18]. The EURAMOS-1 study reported that those patients, who had a poor histological response to neoadjuvant chemotherapy, were associated with worse survival outcome after surgery [5].

As previously reported, the patients with lung metastasis showed poor survival [7, 19, 20]. In the present study, the average overall survival of SLM patients, MLM patients and patients without lung metastasis were 33.3 ± 12.6, 45.8 ± 7.4 and 139.2 ± 2.8 months, respectively. The treatment of pulmonary metastatic lesions showed significant effect on the improved survival of osteosarcoma patients. For osteosarcoma patients with resectable lung metastasis, the NCCN guidelines recommend wide excision of the primary tumor and preoperative chemotherapy should be performed [10]. Meanwhile, pulmonary metastasectomy should be under the consideration in selected cases. It was reported that patients with less lesions, unilateral lung disease and patients after metastasectomy showed improved survival [21, 22]. In our study, most patients with lung metastasis were offered chemotherapy instead of lung surgery. Gemcitabine, docetaxel and other new agents, including regorafenib [23] and apatinib [24], can be potential second-line choices. With the accurate prediction of survival and benefit from metastasectomy on lung function improvement, the metastasectomy should be encouraged in the eligible patients.

Twenty-one (10.3%) patients in the current study showed lung metastasis at the diagnosis of osteosarcoma, which was less than that previously reported [8, 25]. T3 stage and the presence of bone metastasis were risk factors for synchronous lung metastasis in our study, which was consistent with previous results from SEER [8]. During the median follow-up time of 49 months, 67 patients (33%) were detected with lung metastasis. The average interval time from osteosarcoma to lung metastasis was 14.0 ± 14.1 months. Accordance with a previous study, most of lung metastasis happened in the first two or three years [26]. The proportion of patients with lung metastasis was 58.7% and 28.6% in the first and second year after osteosarcoma diagnosis, respectively. Thus, lung CT should be scheduled with high frequency in the first two years. In our current study, the presence of bone metastasis, bad necrosis rate, elevated Ki-67 and LDH were risk factors associated with higher odds of metachronous lung metastasis. And patients with the risk factors should be paid with more attention. A previous study found more lung metastases and bilateral lesions in patients after only surgery of primary tumor, compared with those after surgery plus chemotherapy [6]. Based on different risk of lung metastasis, lung CT plan can be more efficient.

Some limitations should be mentioned. Due to the long internal from osteosarcoma diagnosis, some patients were lost and cannot be reached. Limited size of the included patients and unknown information in some variables caused uncertainty in data statistics. For example, the assessment of HUVOS necrosis rate after neoadjuvant chemotherapy were unavailable in the majority of patients. Furthermore, the limitation of the retrospective study design also leads to weakness to draw confirming conclusions.

## Conclusions

In summary, the osteosarcoma patients in our institute were effectively treated, with the 5-year overall survival of 70%. The incidences of synchronous and metachronous lung metastasis were 10.3%, and 33%, respectively. The prognostic factors found in the current study can be significant on survival prediction. Risk factors of lung metastasis can be used to identify high-risk patients and guide individualized screening.

## Supplementary Information

**Additional file 1:** **Supplementary Table 1.** The definitions of T stage and N stage in patients with osteosarcoma.

**Additional file 2:** **Supplementary Table 2.** Identification of the prognostic factors in osteosarcoma patients with lung metastasis (N=50).

**Additonal file 3:** **Supplementary Table 3.** The detailed information of osteosarcoma patients who underwent pulmonary metastasectomy.

## Data Availability

The datasets used in the current study can be accessed from the corresponding author on reasonable request.

## List of abbreviations

SEER: Surveillance, Epidemiology and End Results
CT: Computerized tomography
ALP: Alkaline phosphatase
LDH: Lactic dehydrogenase
OS: Overall survival
SLM: Synchronous lung metastasis
MLM: Metachronous lung metastasis
CI: Confidence interval
HR: Hazard ratio
OR: Odds ratio
