# Supplementary material for: Incidence, survival, and associated factors estimation in osteosarcoma patients with lung metastasis: a single-center experience of 11 years in Tianjin, China
Source: BMC Cancer. 2023 Jun 5;23:506. doi: 10.1186/s12885-023-11024-9 (PMC10240748; doi:10.1186/s12885-023-11024-9)
Supplement: Supplementary file 1 — Additional file 1: Supplementary Table 1. The definitions of T stage and N stage in patients with osteosarcoma. [file 12885_2023_11024_MOESM1_ESM.docx]

**Supplementary Table 1. The definitions of T stage and N stage in patients with osteosarcoma.**

**T stage**

| **Tumors Located in Appendicular Skeleton, Trunk, Skull, and Facial Bones** | |
| --- | --- |
| TX | Primary tumor cannot be assessed |
| T0 | No evidence of primary tumor |
| T1 | Tumor ≤8 cm in greatest dimension |
| T2 | Tumor >8 cm in greatest dimension |
| T3 | Discontinuous tumors in the primary bone site |
| **Tumors Located in Spine** | |
| TX | Primary tumor cannot be assessed |
| T0 | No evidence of primary tumor |
| T1 | Tumor to one vertebral segment or two adjacent vertebral segments |
| T2 | Tumor confined to three adjacent vertebral segments |
| T3 | Tumor confined to four or more adjacent vertebral segments, or any nonadjacent vertebral segments |
| T4 | Extension into the spinal canal or great vessels |
| T4a | Extension into the spinal canal |
| T4b | Evidence of gross vascular invasion or tumor thrombus in the great vessels |

| **Tumors Located in Pelvis** | |
| --- | --- |
| TX | Primary tumor cannot be assessed |
| T0 | No evidence of primary tumor |
| T1 | Tumor confined to one pelvic segment with no extraosseous extension |
| T1a | Tumor ≤8 cm in greatest dimension |
| T1b | Tumor >8 cm in greatest dimension |
| T2 | Tumor confined to one pelvic segment with extraosseous extension or two segments without extraosseous extension |
| T2a | Tumor ≤8 cm in greatest dimension |
| T2b | Tumor >8 cm in greatest dimension |
| T3 | Tumor spanning two pelvic segments with extraosseous extension |
| T3a | Tumor ≤8 cm in greatest dimension |
| T3b | Tumor >8 cm in greatest dimension |
| T4 | Tumor spanning three pelvic segments or crossing the sacroiliac joint |
| T4a | Tumor involves sacroiliac joint and extends medial to the sacral neuroforamen |
| T4b | Tumor encasement of external iliac vessels or presence of gross tumor thrombus in major  pelvic vessels |

**N stage**

| **Regional Lymph Nodes** | |
| --- | --- |
| NX | Regional lymph nodes cannot be assessed |
| N0 | No regional lymph node metastasis |
| N1 | Regional lymph node metastasis |
