# Supplementary material for: Incidence, survival, and associated factors estimation in osteosarcoma patients with lung metastasis: a single-center experience of 11 years in Tianjin, China
Source: BMC Cancer. 2023 Jun 5;23:506. doi: 10.1186/s12885-023-11024-9 (PMC10240748; doi:10.1186/s12885-023-11024-9)
Supplement: Supplementary file 2 — Additional file 2: Supplementary Table 2. Identification of the prognostic factors in osteosarcoma patients with lung metastasis (N=50). [file 12885_2023_11024_MOESM2_ESM.docx]

**Supplementary Table 2. Identification of the prognostic factors in osteosarcoma patients with lung metastasis (N=50).**

| **Variables** | **Univariate** | | **Multivariate** | |
| --- | --- | --- | --- | --- |
|  | **HR (95% CI)** | ***P value*** | **HR (95% CI)** | ***P value*** |
| Age (year) |  |  |  |  |
| ≤18 | 1.00 (Reference) |  |  |  |
| 19-40 | 0.716 (0.338-1.518) | *0.383* |  |  |
| ≥41 | 1.560 (0.526-4.625) | *0.422* |  |  |
| Gender |  |  |  |  |
| Male | 1.00 (Reference) |  |  |  |
| Female | 0.784 (0.378-1.626) | *0.513* |  |  |
| Tumor site |  |  |  |  |
| Upper limb | 1.00 (Reference) |  |  |  |
| Lower limb | 0.805 (0.279-2.323) | *0.688* |  |  |
| Spine-pelvis | 0.832 (0.146-4.737) | *0.836* |  |  |
| Stage T |  |  |  |  |
| T1 | 1.00 (Reference) |  | 1.00 (Reference) |  |
| T2 | 0.853 (0.419-1.738) | *0.662* | 0.536 (0.227-1.267) | *0.156* |
| T3 | 1.046 (0.134-8.172) | *0.966* | 3.074 (0.226-41.805) | *0.399* |
| Unknown | NA | *NA* | NA | *NA* |
| Stage N |  |  |  |  |
| N0 | 1.00 (Reference) |  |  |  |
| N1 | 1.360 (0.403-4.589) | *0.620* |  |  |
| Unknown | 1.623 (0.694-3.795) | *0.264* |  |  |
| Bone Mets |  |  |  |  |
| No | 1.00 (Reference) |  | 1.00 (Reference) |  |
| Yes | 1.136 (0.569-2.268) | *0.719* | 1.147 (0.459-2.869) | *0.769* |
| Lung Mets |  |  |  |  |
| SLM | 1.00 (Reference) |  | 1.00 (Reference) |  |
| MLM | 1.378 (0.419-4.528) | *0.598* | 2.616 (0.448-15.263) | *0.285* |
| Surgery |  |  |  |  |
| No | 1.00 (Reference) |  | 1.00 (Reference) |  |
| Salvage | 0.385 (0.104-1.432) | *0.155* | 0.702 (0.116-4.232) | *0.699* |
| Amputation | 0.811 (0.232-2.831) | *0.742* | 2.181 (0.348-13.680) | *0.405* |
| Unknown | NA | *NA* | NA | *NA* |
| Chemotherapy |  |  |  |  |
| Yes | 1.00 (Reference) |  | 1.00 (Reference) |  |
| Unknown | 0.520 (0.071-3.821) | *0.52* | 78.032 (2.958-2058.190) | *0.009* |
| Necrosis |  |  |  |  |
| <90% | 1.00 (Reference) |  | 1.00 (Reference) |  |
| ≥90% | 2.280 (0.297-17.505) | *0.428* | 4.590 (0.482-43.701) | *0.185* |
| Unknown | 1.022 (0.511-2.045) | *0.951* | 1.539 (0.693-3.420) | *0.290* |
| Ki-67 |  |  |  |  |
| <50% | 1.00 (Reference) |  |  |  |
| ≥50% | 1.186 (0.282-4.997) | *0.816* |  |  |
| Unknown | 1.664 (0.492-5.623) | *0.413* |  |  |
| LDH |  |  |  |  |
| Normal | 1.00 (Reference) |  |  |  |
| Elevated | 0.765 (0.356-1.643) | *0.492* |  |  |
| ALP |  |  |  |  |
| Normal | 1.00 (Reference) |  | 1.00 (Reference) |  |
| Within one time | 0.870 (0.330-2.298) | *0.779* | 0.488 (0.148-1.606) | *0.238* |
| More than two times | 1.258 (0.572-2.769) | *0.568* | 1.015 (0.395-2.607) | *0.976* |

**Abbreviations**: NA=Not available; HR=Hazard ratio; CI=Confidence interval; Mets=Metastases; SLM=Synchronous lung metastasis; MLM =Metachronous lung metastasis; ALP=Alkaline phosphatase; LDH=Lactate dehydrogenase.
