# Supplementary material for: Incidence, survival, and associated factors estimation in osteosarcoma patients with lung metastasis: a single-center experience of 11 years in Tianjin, China
Source: BMC Cancer. 2023 Jun 5;23:506. doi: 10.1186/s12885-023-11024-9 (PMC10240748; doi:10.1186/s12885-023-11024-9)
Supplement: Supplementary file 3 — Additonal file 3: Supplementary Table 3. The detailed information of osteosarcoma patients who underwent pulmonary metastasectomy. [file 12885_2023_11024_MOESM3_ESM.docx]

**Supplementary Table 3. The detailed information of osteosarcoma patients who underwent pulmonary metastasectomy.**

| **Age** | **Gender** | **Tumor site** | **Historical types** | **T stage** | **Primary tumor surgery** | **Preoperative chemotherapy** | **SLM/**  **MLM** | **Interval between osteosarcoma and MLM (months)** | **Laterality** | **Number of metastatic nodules** | **Size of metastatic nodules (cm)** | **Status** | **OS**  **(months)** |
| --- | --- | --- | --- | --- | --- | --- | --- | --- | --- | --- | --- | --- | --- |
| 11 | Female | Tibia | Conventional | T2 | Salvage | MAP* | MLM | 11 | Unilateral | 1 | 2.3×2.1 | Alive | 103 |
| 20 | Male | Ulna | Conventional | T2 | Salvage | MAP | MLM | 14 | Unilateral | 1 | 2.6×1.7 | Alive | 115 |
| 10 | Female | Femur | Conventional | T1 | Salvage | MAP | MLM | 19 | Unilateral | 2 | 1.9×1.7  1.9×1.5 | Dead | 53 |
| 14 | Female | Tibia | Conventional | T2 | Amputation | MAP | MLM | 24 | Unilateral | 1 | 2.1×1.8 | Dead | 43 |
| 14 | Female | Femur | Conventional | T2 | Amputation | MAP | MLM | 31 | Bilateral | 3 | 3.1×2.8  2.5×1.6  2.3×1.8 | Loss to follow up | |

*MAP=cisplatin, doxorubicin, and high-dose methotrexate.
